# Ultra-Low Thermal Conductivity and Improved Thermoelectric Performance in Tungsten-Doped GeTe

**DOI:** 10.3390/nano14080722

**Published:** 2024-04-20

**Authors:** Zhengtang Cai, Kaipeng Zheng, Chun Ma, Yu Fang, Yuyang Ma, Qinglin Deng, Han Li

**Affiliations:** 1 School of Physics and Materials Science, Guangzhou University, Guangzhou 510006, China; zt.caine@e.gzhu.edu.cn (Z.C.); 2112119106@e.gzhu.edu.cn (K.Z.); 2112119081@e.gzhu.edu.cn (C.M.); 2112219110@e.gzhu.edu.cn (Y.F.); 32119600037@e.gzhu.edu.cn (Y.M.); 2 Research Center for Advanced Information Materials (CAIM), Huangpu Research & Graduate School of Guangzhou University, Guangzhou 510555, China

**Keywords:** GeTe, ultra-low thermal conductivity, synergistic effects, multiscale materials, electronic engineering

## Abstract

Compared to SnTe and PbTe base materials, the GeTe matrix exhibits a relatively high Seebeck coefficient and power factor but has garnered significant attention due to its poor thermal transport performance and environmental characteristics. As a typical p-type IV–VI group thermoelectric material, W-doped GeTe material can bring additional enhancement to thermoelectric performance. In this study, the introduction of W, Ge_1−*x*_W*_x_*Te (*x* = 0, 0.002, 0.005, 0.007, 0.01, 0.03) resulted in the presence of high-valence state atoms, providing additional charge carriers, thereby elevating the material’s power factor to a maximum PF_peak_ of approximately 43 μW cm^−1^ K^−2^, while slightly optimizing the Seebeck coefficient of the solid solution. Moreover, W doping can induce defects and promote slight rhombohedral distortion in the crystal structure of GeTe, further reducing the lattice thermal conductivity *κ_lat_* to as low as approximately 0.14 W m^−1^ K^−1^ (*x* = 0.002 at 673 K), optimizing it to approximately 85% compared to the GeTe matrix. This led to the formation of a p-type multicomponent composite thermoelectric material with ultra-low thermal conductivity. Ultimately, W doping achieves the comprehensive enhancement of the thermoelectric performance of GeTe base materials, with the peak ZT value of sample Ge_0.995_W_0.005_Te reaching approximately 0.99 at 673 K, and the average ZT optimized to 0.76 in the high-temperature range of 573–723 K, representing an increase of approximately 17% compared to pristine GeTe within the same temperature range.

## 1. Introduction

Thermoelectric (TE) materials have attracted considerable attention as environmentally friendly and green energy materials due to their ability to directly convert thermal energy into electrical energy. The performance of TE materials is typically assessed using the dimensionless figure of merit ZT, defined as ZT = *S^2^σT*/*κ_tot_*, where *S* represents the Seebeck coefficient, *σ* is the electrical conductivity, *T* is the temperature in Kelvin, and *κ_tot_* denotes the total thermal conductivity (the sum of the lattice thermal conductivity and electronic thermal conductivity, i.e., *κ_tot_* = *κ_lat_* + *κ_e_*). The optimization of TE material performance primarily involves tuning the carrier concentration to enhance the electrical transport properties and improving the phonon transport characteristics to reduce the lattice thermal conductivity [1,2,3], thereby increasing the power factor and ultimately enhancing the thermoelectric performance of the material [4,5].

In recent years, GeTe has emerged as a typical IV–VI group semiconductor thermoelectric material [6]. Benefiting from its crystal structure closely resembling that of SnTe [7,8,9] and PbTe [10,11,12], GeTe exhibits a relatively high intrinsic Seebeck coefficient. However, due to the environmental toxicity of PbTe, poor mechanical and thermoelectric properties of SnTe, and the fact that the peak ZT value of GeTe-based materials can exceed 0.8 in the temperature range of 300–750 K, GeTe has become a hot topic in thermoelectric research. Nevertheless, the intrinsic electrical properties of GeTe are generally suboptimal, its thermal stability is inadequate, and its thermal transport performance is poor, limiting its widespread application. GeTe possesses a narrow bandgap structure and exhibits different crystal structures at different temperatures: it adopts a rhombohedral crystal structure (r-GeTe) at room temperature and a cubic crystal structure (c-GeTe) at high temperatures, with a phase transition from r- to c-GeTe occurring around ~700 K [3,13]. We also realized that a slight rhombohedral distortion occurs along the [1 1 1] crystal axis direction [14], which results in the convergence of the valence band [8,12,15]. Nevertheless, the phase transition is detrimental to the thermoelectric properties of GeTe materials as it likely results in the additional loss of electron energy due to extensive lattice deformation. This, in turn, negatively impacts carrier mobility, affecting the figure of merit (ZT). Essentially, the primary goal of thermoelectric material optimization is to increase the ZT value, which can be achieved by reasonably regulating the coupling relationship between the three thermoelectric performance parameters: *S*, *σ*, and *κ_tot_*. When the electrical conductivity of the sample is increased, its electronic thermal conductivity also increases. However, excessively high electrical conductivity has a detrimental impact on carrier mobility, resulting in a decreasing Seebeck coefficient and affecting the thermoelectric properties of the material. Therefore, the key to improving the ZT value of materials lies in synergistically regulating the electrical and thermal transport properties. In order to optimize the thermoelectric properties of GeTe-based materials, researchers typically employ doping or alloying techniques. In doping engineering, the carrier concentration and energy band structure of the material can be optimized by applying donor doping, which results in a larger Seebeck coefficient and a higher power factor. Furthermore, doping engineering can result in the introduction of point defects, dislocations, and grain boundary defects, which lead to lattice distortions in GeTe material. This intensifies phonon scattering within the material and reduces the lattice’s thermal conductivity, thereby further increasing the ZT value.

In our previous work, it was demonstrated that tungsten (W) doping can effectively enhance the electrical conductivity of the material, resulting in the optimization of a higher power factor and the capacity to elevate the Seebeck coefficient [16]. Regarding thermal transport properties, previous research has demonstrated that W doping can also introduce a multitude of grain boundary and dislocation defects, a considerable number of which can enhance phonon scattering in the material, resulting in a significant reduction in the lattice thermal conductivity and, ultimately, a dramatic optimization of the ZT value.

Consequently, the present study aims to achieve the same optimization via the elemental tungsten (W) doping of a GeTe system [17]. It is noteworthy that the electrical and thermal transport properties of GeTe-based materials can be modulated by designing W substitution (donor doping). Firstly, the intervention of W substitution introduced high-valence atoms, resulting in an increase in the power factor of the samples across the entire temperature range due to the introduction of additional carriers. Secondly, W also exists in the solid solution of Ge_1-*x*_W*_x_*Te (*x* = 0, 0.002, 0.005, 0.007, 0.01, 0.03) in the form of W atoms [18,19,20], forming defects to replace some of the Ge vacancies [21,22,23]. On the other hand, the formation of defects further strengthens the scattering of phonons in the material [24,25,26], significantly reducing the lattice thermal conductivity. Ultimately, we achieved a significant improvement in the peak power factor (PF_peak_) at 673 K up to ~43 μW cm^−1^ K^−2^ and the optimization of the ultra-low lattice thermal conductivity of 0.14 W m^−1^ K^−1^ at 673 K for a W doping concentration of *x* = 0.002. This contributes to a remarkable enhancement in the average ZT value up to ~0.76 within the temperature range of 573–723 K and represents a 17% increase compared to the GeTe matrix, thereby improving the thermoelectric performance of GeTe-based materials overall [7,9,27].

## 2. Materials and Methods

### 2.1. Materials and Synthesis

This study involved the preparation of Ge_1−*x*_W*_x_*Te (*x* = 0, 0.002, 0.005, 0.007, 0.01, 0.03) compounds in an inert gas (Ar) atmosphere within a glovebox. High-purity elements Ge (99.999%, Aladdin, Shanghai, China), W (99.99%, Aladdin), and Te (99.99%, Aladdin) were accurately weighed and transferred into graphite crucibles, thoroughly mixed, and then packed into quartz glass tubes. Subsequently, the tubes were sealed under high vacuum conditions (below 10^−5^ Torr). The loaded quartz tubes were then placed in a box furnace and slowly heated to 1373 K, maintained for 12 h, followed by quenching with water to room temperature. Subsequently, the temperature was raised again to 923 K for annealing over 3 days, and finally slowly cooled to room temperature. The resulting alloy ingots were carefully removed and manually ground into fine powders using an agate mortar and pestle. Appropriate amounts of the ground powders were weighed and loaded into prepared graphite molds, which were then placed into a spark plasma sintering furnace (SPS, KCE-FCT HPD10, Sachsenheim, Germany) for vacuum hot-pressing sintering. During the sintering process, the samples were subjected to a uniaxial pressure of 60 MPa in a high vacuum, heated to 573 K in 5 min, and then heated to 823 K in 3 min. Finally, they were held for 5 min before being removed and allowed to cool naturally.

### 2.2. Measurement and Characterizations

After SPS sintering and subsequent cooling, the samples were subjected to wire cutting to produce rectangular prismatic specimens measuring 12 × 3 × 3 mm. These specimens were then lightly polished and placed into a four-probe thermoelectric measurement system (CTA, CRYOALL CTA-3S, Beijing, China) to simultaneously measure the temperature-dependent electrical conductivity σ and Seebeck coefficient *S*. Additionally, the thin discs obtained from SPS sintering underwent thermal diffusivity testing using the Laser-Flash Thermal Conductivity Instrument (LFA 467 HT NETZSCH, Tannesstein, Germany) to determine the thermal diffusivity coefficient *D* of the samples. Subsequently, the density *ρ* of each sample was determined using the Archimedes’ displacement method. The total thermal conductivity *κ_tot_* of the samples was calculated using the formula *κ_tot_* = *DρC_p_*, where *C_p_* represents the theoretical specific heat capacity of the GeTe material. Furthermore, the electronic thermal conductivity *κ_e_* of the samples was calculated according to the Wiedemann–Franz law and the formula *κ_e_* = *LσT*. Finally, the lattice thermal conductivity *κ_lat_* of the samples was obtained by subtracting *κ_e_* from *κ_tot_*. Subsequently, X-ray diffraction (XRD) analysis was conducted at room temperature using a Rigaku Smartlab 9 KW (Tokyo, Japan) instrument to perform the compositional analysis of the samples. To further understand the influence of the microstructural composition of the samples on the thermoelectric properties of the GeTe materials, a transmission electron microscopy (TEM, FEI HELIOS 5CX, Hillsboro, OR, USA) characterization test was performed to observe and analyze the mechanism.

## 3. Results and Discussion

**Figure 1:**
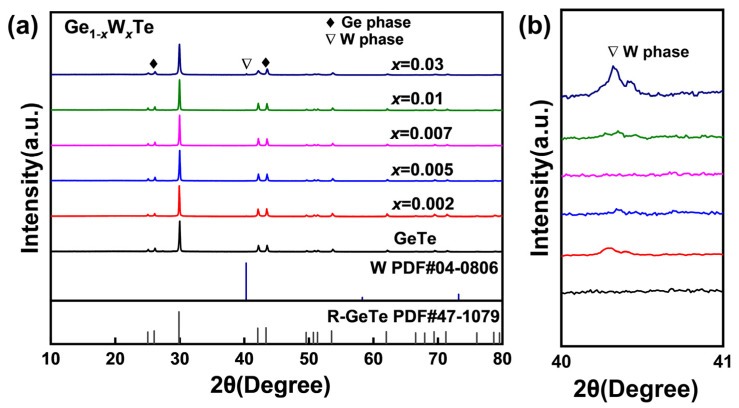
(**a**) Room temperature powder XRD patterns of the Ge_1−*x*_W*_x_*Te (*x* = 0, 0.002, 0.005, 0.007, 0.01, 0.03) samples; (**b**) enlarged view of the W phase at 40~41°.

**Figure 2:**
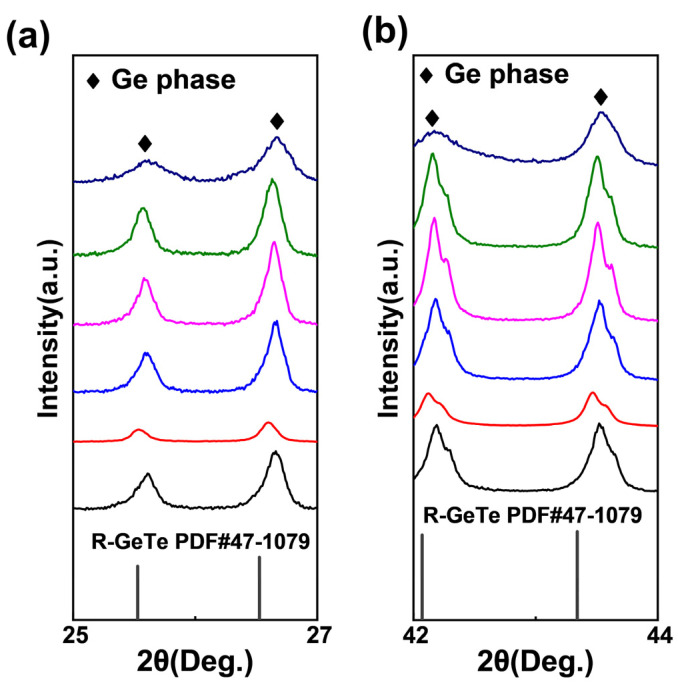
Room temperature powder XRD patterns enlarged view of the Ge phase of the Ge_1−*x*_W*_x_*Te (*x* = 0, 0.002, 0.005, 0.007, 0.01, 0.03) samples (**a**) at 25~27°; (**b**) enlarged view at 42~44°.

The room temperature powder X-ray diffraction (XRD) results of the Ge_1−*x*_W*_x_*Te samples (*x* = 0, 0.002, 0.005, 0.007, 0.01, 0.03) are depicted in Figure 1a. It can be observed that upon W doping into GeTe, most of the diffraction peaks match well with the rhombohedral phase single-phase structure of r-GeTe (space group: *R*3*m*, PDF: 47-1079). In Figure 2a,b, the slight precipitation of Ge can be observed at low angles of 25~26° and 42~44°, respectively. This phenomenon is commonly observed throughout the entire GeTe system and is attributed to the presence of large Ge vacancies in the GeTe system, consistent with previous reports [3,28,29], which do not significantly affect the ultimate thermoelectric performance of GeTe-based materials [2,24,30]. The appearance of dual peaks of GeTe at this angle further confirms its rhombohedral structure at room temperature. Moreover, the sample Ge_0.97_W_0.03_Te exhibits a distinct second-phase diffraction peak at ~40° in Figure 1b. Through XRD spectrum analysis and data comparison, the main component of the diffraction peak at this angle is identified as W (space group: *Im*3*m*, PDF: 04-0806), indicating a solubility of W in the GeTe system of approximately 0.5 mol%. Additionally, GeTe undergoes a phase transition from the r- to the c-GeTe (c-GeTe, space group: *Fm*-3*m*) at ~700 K, transforming its crystal structure along the [1 1 1] direction into a cubic structure, which is similar to that of PbTe [31] and SnTe [1,20]. This cubic structure is also similar to the crystal structure of the precipitated W phase in this study, suggesting structural similarity with GeTe [32].

**Figure 3:**
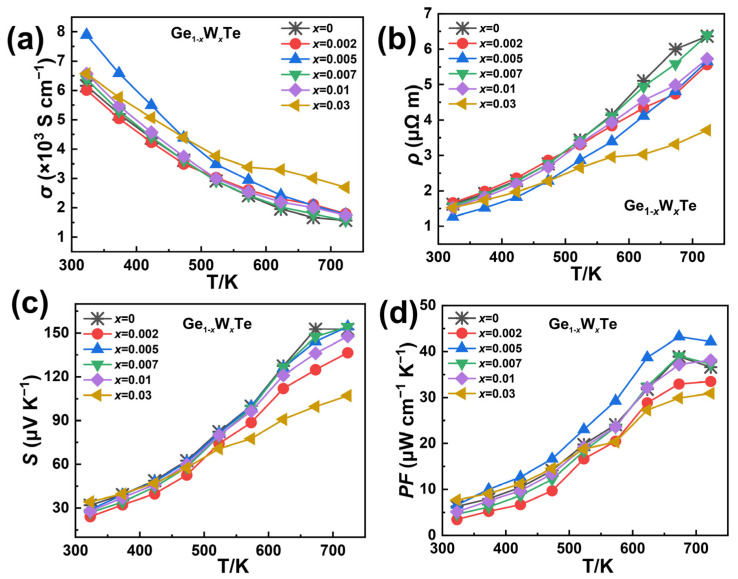
Temperature-dependent electrical performance of the Ge_1−_*_x_*W*_x_*Te (*x* = 0, 0.002, 0.005, 0.007, 0.01, 0.03) solid solution; (**a**) electrical conductivity *σ*; (**b**) resistivity *ρ*; (**c**) Seebeck coefficient *S*; (**d**) power factor PF.

Upon W doping in GeTe, the electrical transport properties of the material undergo subtle changes. In Figure 3a, as the proportion of W doping increases, the electrical conductivity (*σ*) exhibits a trend of initially decreasing and then increasing. Compared to the GeTe matrix, when *x* = 0.002, the *σ* of the sample Ge_0.998_W_0.002_Te decreases to ~1.8 × 10^3^ S cm^−1^ at 723 K. When *x* > 0.005, the *σ* of the sample Ge_1−*x*_W*_x_*Te increases with the amount of W doping, and at *x* = 0.03, the *σ* of the sample Ge_0.97_W_0.03_Te is higher than that of other samples and higher than the GeTe matrix overall. This may be attributed to the introduction of the high-valence state W into GeTe, providing additional charge carriers and resulting in an increase in the carrier concentration. In Figure 3c, a schematic diagram of the Seebeck coefficient (*S*) of the sample Ge_1−*x*_W*_x_*Te (*x* = 0, 0.002, 0.005, 0.007, 0.01, 0.03) as a function of temperature is presented. In the mid-low temperature range (300–573 K), the *S* of the sample Ge_0.995_W_0.005_Te shows a certain improvement, reaching ~154 μV K^−1^ at 723 K. When *x* ≥ 0.005, *S* begins to decline, especially when *x* = 0.03, the sample Ge_0.97_W_0.03_Te exhibits a significant reduction in *S* in the high-temperature range (600–750 K). This may be because W has reached its solubility limit in the GeTe system, existing in the material structure in the form of W. Additionally, as W belongs to heavy atoms, it affects carrier mobility. In Figure 3d, the power factor PF of the sample Ge_0.998_W_0.002_Te is lower than that of the undoped sample GeTe, and when the doping level rises to *x* = 0.005, the PF of the sample Ge_0.995_W_0.005_Te is higher than that of the undoped GeTe matrix, with a peak value reaching ~43 μW cm^−1^ K^−2^. When the W doping ratio is greater than *x* = 0.005, the PF of the sample Ge_1−*x*_W*_x_*Te decreases with the increase in the doping concentration. This phenomenon occurs because W doping introduces additional heavy atoms into the GeTe material, significantly affecting the electrical transport properties of the sample [5,33,34]. Despite the weak increase in the Seebeck coefficient of the sample Ge_0.995_W_0.005_Te, W doping leads to a higher peak PF in the material compared to the GeTe matrix.

**Figure 4:**
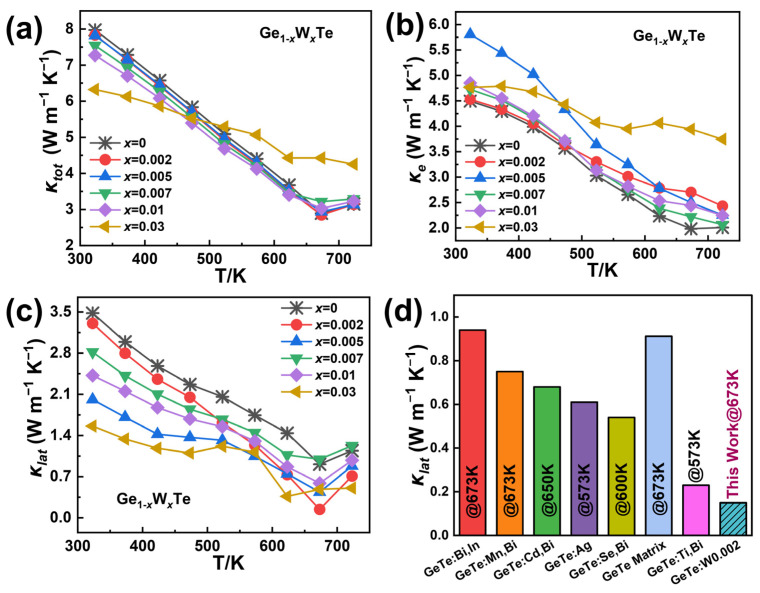
Thermal transport properties of the Ge_1−*x*_W*_x_*Te (*x* = 0, 0.002, 0.005, 0.007, 0.01, 0.03) samples; (**a**) total thermal conductivity *κ_tot_*; (**b**) electronic thermal conductivity *κ_e_*; (**c**) lattice thermal conductivity *κ_lat_*; (**d**) lattice thermal conductivity of this work compared to the GeTe matrix; GeTe: Bi, In [35]; GeTe: Mn, Bi [13]; GeTe: Cd, Bi [36]; GeTe: Ag [37]; GeTe: Se, Bi [38]; and GeTe: Ti, Bi [39].

In Figure 4a, for the Ge_1−*x*_W*_x_*Te samples (*x* = 0, 0.002, 0.005, 0.007, 0.01, 0.03), at *x* = 0.002, the total thermal conductivity *κ_tot_* exhibits a slight decrease compared to the undoped sample, while at *x* = 0.005, the change in *κ_tot_* of the sample Ge_0.995_W_0.005_Te is minimal, differing only marginally from the sample at *x* = 0.002. As the doping ratio continues to increase, the overall trend of *κ_tot_* continues to decrease. At *x* = 0.03, the *κ_tot_* of the sample Ge_0.97_W_0.03_Te remains relatively flat, and thermal performance deteriorates. This phenomenon is consistent with the earlier XRD findings, where the degradation is attributed to the different forms of W presence in the GeTe system, which significantly affects the carriers, ultimately influencing the thermal transport properties. Additionally, due to the higher valence state of W compared to Ge^2+^, it may introduce additional carriers into the system, further increasing the carrier concentration of the GeTe base material, resulting in a higher electronic thermal conductivity *κ_e_* compared to undoped GeTe, with higher proportions of W leading to higher *κ_e_* and a flattening trend. In Figure 4b, the slope of the *κ_e_* curve for the sample Ge_0.97_W_0.03_Te decreases, becoming flatter overall. When the W doping ratio reaches *x* = 0.005, the *κ_e_* of the sample Ge_0.995_W_0.005_Te reaches ~5.8 W m^−1^ K^−1^ at temperatures near room temperature to 323 K, 29% higher than other samples with different W doping compositions. In Figure 4c, due to the generally higher electronic thermal conductivity *κ_e_* of the Ge_1−*x*_W*_x_*Te samples compared to the undoped GeTe base material, the lattice thermal conductivity *κ_lat_* of the samples decreases significantly. For instance, the *κ_lat_* of the sample Ge_0.998_W_0.002_Te reaches a minimum value of ~0.14 W m^−1^ K^−1^ at 673 K, while the *κ*_lat_ of the sample Ge_0.995_W_0.005_Te decreases to ~0.44 W m^−1^ K^−1^ at 673 K. The effective reduction of *κ_lat_* in the material is achieved via appropriate W doping into the GeTe system.In Figure 4d, the extremely low *κ_lat_* of the sample Ge_0.998_W_0.002_Te reduces by approximately 85%, 81%, 79%, 77%, 74%, and 39% compared to the works of Yang et al. on GeTe: Bi, In [35], Liu et al. on GeTe: Mn, Bi [13], Li et al. on GeTe: Cd, Bi [36], Srinivasan et al. on GeTe: Ag [37], Xu et al. on GeTe: Se, Bi [38], and Shuai et al. on GeTe: Ti, Bi [39], respectively.

**Figure 5:**
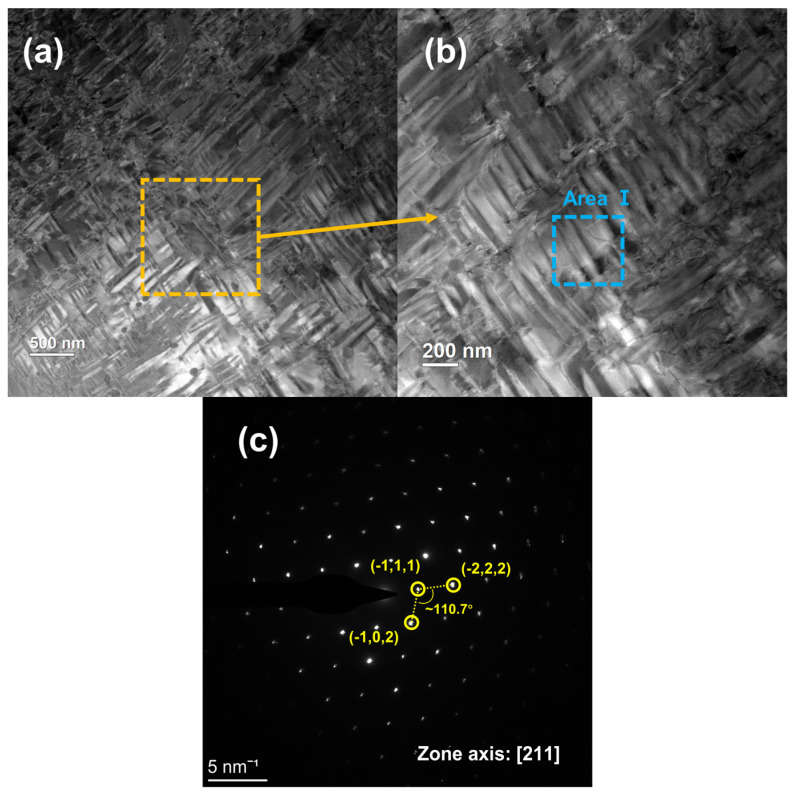
Transmission scanning microscopy (TEM) images of the Ge_0.995_W_0.005_Te solid solution: (**a**) low magnification TEM image; (**b**) enlarged TEM image of the area surrounded by dashed lines in (**a**); (**c**) selected area electron diffraction (SAED) pattern of the GeTe matrix with the [2 1 1] zone axis.

In order to further investigate the mechanism of the influence of W doping on the thermoelectric transport properties of the GeTe system and the microstructural morphology of the material, we performed a transmission electron microscopy (TEM) characterization test on the Ge_0.995_W_0.005_Te sample. As shown in Figure 5a, a low magnification TEM image of the sample Ge_0.995_W_0.005_Te is demonstrated, and the typical herringbone fishbone structure of the GeTe material can be clearly observed [40], which is uniformly interlaced throughout the sample. The magnified image of the orange dashed box area in Figure 5a corresponds to that shown in Figure 5b, and the high-resolution image can better illustrate the herringbone fishbone structure, from which it can be seen that the W doping of GeTe does not produce a second phase to the microstructure of the material. It also improves the orderliness of the atomic arrangement of the GeTe material. Subsequently, selected area electron diffraction (SAED) was performed on the same sample by choosing the appropriate region. The SAED image of the Ge_0.995_W_0.005_Te sample at high magnification along the direction of the [2 1 1] crystal axis is demonstrated in Figure 5c, from which the diffractograms show that the synthesized sample has a single-crystalline structure and is consistent with the r-GeTe crystalline phase. The angle between the ($\bar{1}$,1,1), ($\bar{2}$,2,2), and ($\bar{1}$,0,2) crystal planes is ~110.7°, which is slightly smaller than the 120° angle of the GeTe rhombic phase structure, which precisely indicates that the W doping is introduced to more likely substitute the Ge sites and bring about slight nanoscale lattice distortions [41], which agrees with the results of the sample’s electrical conductivity test in Figure 3d.

**Figure 6:**
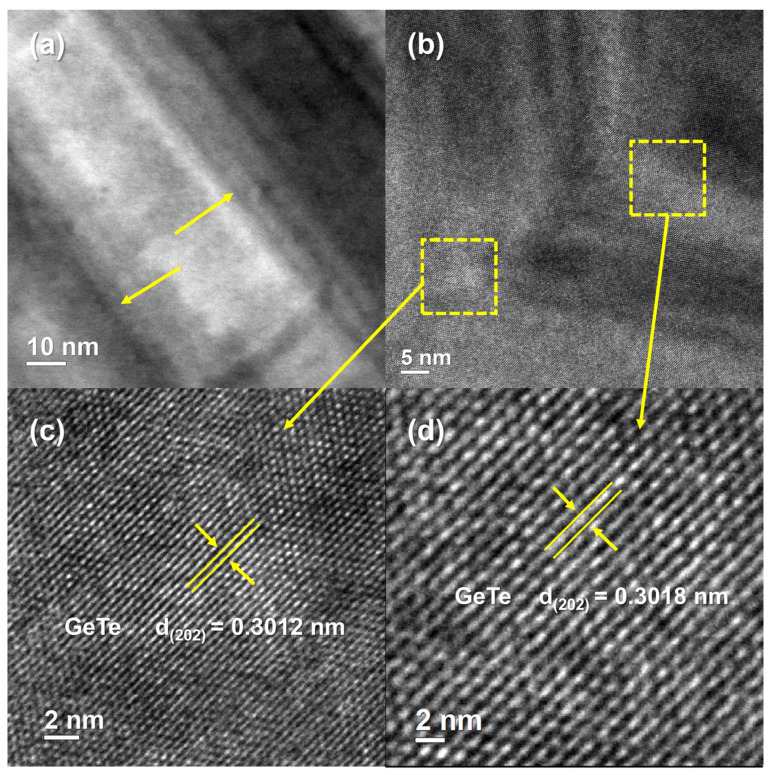
(**a**) High magnification TEM image of the blue dashed box area (area I) corresponding to Figure 5b of the Ge_0.995_W_0.005_Te solid solution; (**b**) HRTEM image of the same solid solution; (**c**,**d**) enlarged HRTEM images corresponding to the area surrounded by yellow dashed lines in (**a**), respectively.

The blue dashed box area in Figure 5b of the Ge_0.995_W_0.005_Te sample was selected to be photographed at high magnification to obtain a high-resolution magnified image, as shown in Figure 6a. It clearly demonstrates that the Ge_0.995_W_0.005_Te sample exhibits a pronounced dislocation structure in Figure 6a, resulting from the disparate mass and size of the Ge and W atoms. This leads to the typical lattice distortions observed in W doping at the Ge sites, which introduces defects, optimizes the electron–phonon transport properties of the material, and further enhances the phonon scattering. This is consistent with the findings of our previous work [16], which demonstrated that W is capable of introducing a significant number of defects, thereby greatly reducing the lattice thermal conductivity of the material. In the high-resolution image of Figure 6b, the area within the yellow dashed box represents the interplanar spacing measured for sample Ge_0.995_W_0.005_Te, as indicated by the yellow arrows corresponding to Figure 6c,d, which represent the d spacing values of *d*_(202)_~0.3012 nm and *d*_(202)_~0.3018 nm, respectively. They are both very close to the standard value of the d spacing of the r-GeTe (202) planar of 2.991 Å. This implies that W doping does not bring about a significant second phase but rather modulates the microstructure of the GeTe material by introducing defects. It is noteworthy that these results are in agreement with the previously mentioned XRD and electrical-thermal performance tests, which significantly enhance the electrical conductivity of the GeTe material.

**Figure 7:**
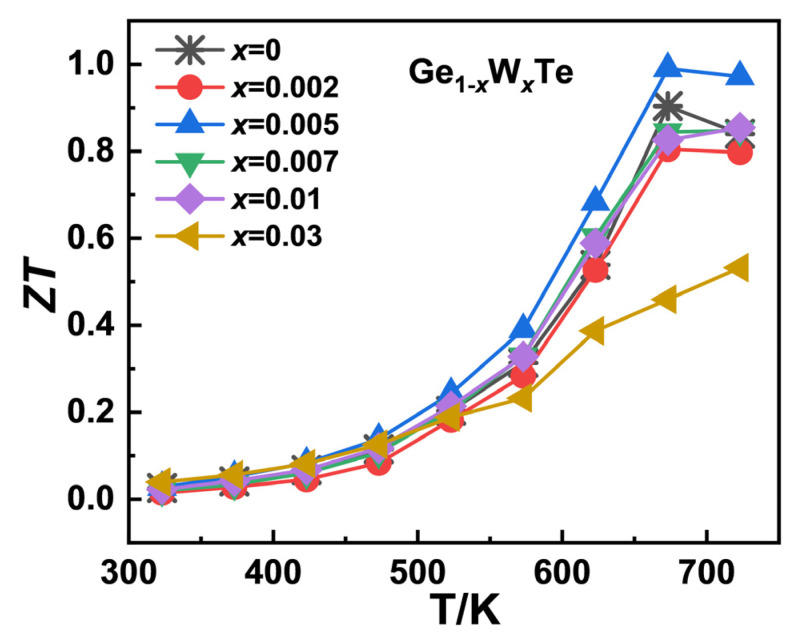
Temperature-dependent trend of the figure of merit (ZT) value of the Ge_1−*x*_W*_x_*Te (*x* = 0, 0.002, 0.005, 0.007, 0.01, 0.03) samples.

Figure 7 depicts the temperature-dependent curves of the sample Ge_1−*x*_W*_x_*Te (*x* = 0, 0.002, 0.005, 0.007, 0.01, 0.03). When the W doping concentration reaches *x* = 0.005, the ZT value of the sample Ge_0.995_W_0.005_Te reaches its maximum, approximately 0.99 at 673 K, 10% higher than the undoped sample. Due to the high melting point of W, in the high-temperature range (573–723 K), the average ZT value of the Ge_1−*x*_W*_x_*Te sample is approximately 0.76, 17% higher than the undoped GeTe matrix at the same temperature range. However, a high dopant concentration (*x* ≥ 0.007) causes an excess of W in the sample, which leads to a decrease in the carrier mobility due to increased carrier scattering; at the same time, the electronic thermal conductivity *κ_e_* increases (as well as the total thermal conductivity *κ_tot_*), leading to a negative optimization in the figure of merit. Therefore, the sample Ge_1−*x*_W*_x_*Te achieves the highest ZT value for the *x* = 0.005 doping concentration.

## 4. Conclusions

In summary, this study elucidates the advantages and disadvantages of W-doped GeTe materials, analyzing the influence of W on the material structure, electrical transport properties, and thermal transport properties. The higher valence state of W allows for the introduction of additional charge carriers, thereby enhancing the electrical conductivity of the material while simultaneously increasing the power factor, thereby improving the Seebeck coefficient. With a higher melting point than GeTe, W stabilizes the rock-salt cubic structure of GeTe (achieved through the phase transition from r-GeTe) near 700 K. It is noteworthy that W doping can also substitute the Ge atoms in the GeTe material system, introducing dislocation defects to optimize the thermal transport performance. W doping can also induce lattice distortions in the GeTe structure [15], enhancing phonon scattering and effectively reducing the lattice thermal conductivity of the material at suitable W doping concentrations, resulting in GeTe-based materials with ultra-low lattice thermal conductivity as low as approximately 0.14 W m^−1^ K^−1^ at 673 K [16,27,42]. Compared to the previous work [35], this study demonstrates a maximum reduction of approximately 85% in lattice thermal conductivity. The experimental results confirmed that W doping not only introduces high-valence state W atoms to enhance the power factor of the sample but also simultaneously reduces the lattice thermal conductivity by optimizing the material structure [32], thereby comprehensively regulating the thermoelectric transport properties of GeTe-based materials. Ultimately, the average ZT value of the sample Ge_0.995_W_0.005_Te reached ~0.76 in the temperature range of 573–723 K, approximately 17% higher than undoped GeTe. Furthermore, the peak ZT value of sample Ge_0.995_W_0.005_Te reached ~0.99 across the entire temperature range, representing a ~10% improvement over the GeTe base material.

## Data Availability

Data are contained within the article.
